# *KRAS* and *BRAF* mutations modify adjuvant chemotherapy outcomes in early stage colorectal cancer

**DOI:** 10.1038/s41698-026-01494-y

**Published:** 2026-05-20

**Authors:** Durgesh Wankhede, Mary Jose Urruchua Rodriguez, Dominic Edelmann, Matthias Kloor, Hendrik Bläker, Alexander Brobeil, Wilfried Roth, Hermann Brenner, Michael Hoffmeister

**Affiliations:** 1 Division of Clinical Epidemiology of Early Cancer Detection, German Cancer Research Center (DKFZ), Heidelberg, Germany; 2 Faculty of Medicine, University of Heidelberg, Heidelberg, Germany; 3 Department of Biostatistics, German Cancer Research Center, Heidelberg, Germany; 4 Department of Applied Tumor Biology, Institute of Pathology, University Hospital Heidelberg, Heidelberg, Germany; 5 Department of General Pathology, Institute of Pathology, Charité University Medicine Hospital, Berlin, Germany; 6 National Center for Tumour Diseases, University Hospital Heidelberg, Heidelberg, Germany; 7 Tissue Bank, National Center for Tumour Diseases, University Hospital Heidelberg, Heidelberg, Germany; 8 Institute of Pathology, University Medical Center Mainz, Mainz, Germany; 9 Cancer Prevention Graduate School, German Cancer Research Center (DKFZ), Heidelberg, Germany

**Keywords:** Cancer, Oncology

## Abstract

*KRAS* and *BRAF*^*V600E*^ mutations are established biomarkers in metastatic colorectal cancer (CRC), but their predictive roles in early-stage disease remain uncertain. We aimed to determine whether *KRAS* and *BRAF*^*V600E*^ mutations modify the association between adjuvant chemotherapy regimen and survival in stage III and high-risk stage II CRC. We analyzed patients who underwent curative resection and molecular profiling for *KRAS* and *BRAF*^*V600E*^. Adjuvant chemotherapy was classified as fluoropyrimidine monotherapy (5FU) or oxaliplatin-based therapy (Ox+). Propensity score overlap weighting addressed non-randomized treatment allocation. Weighted Cox models assessed recurrence-free survival (RFS) and overall survival (OS), including tests for treatment-by-mutation interaction. Among treated patients (*n* = 853), a global interaction test indicated a differential association between regimens and survival by mutation status (OS, *p* = 0.007; RFS *p* = 0.049). Ox+ was associated with improved survival in patients with *KRAS*-mutated tumors (OS HR = 0.68, 95% CI, 0.47–0.98), and a less favorable outcome in *BRAF*-mutated tumors (OS HR = 2.58, 95% CI, 1.16–5.77) compared with 5FU. Outcomes were similar between Ox+ and 5FU in double wild-type (*KRAS* and *BRAF* wild-type) tumors (OS HR = 1.11, 95% CI, 0.82–1.50). These findings suggest molecular heterogeneity in treatment associations that may inform adjuvant therapy selection.

## Introduction

Colorectal cancer (CRC) is a molecularly heterogeneous disease, with clinical outcomes and treatment responses shaped by distinct oncogenic pathways^1^. Central to tumor initiation and progression is the RAS-RAF-MAPK signaling cascade, which governs cell growth and survival^2^. Activating mutations in *KRAS* and *BRAF*, typically mutually exclusive, are among the most common genetic alterations in CRC, occurring in approximately 35–45% and 8–12% of tumors, respectively^2,3^. In metastatic CRC, these mutations are well established as negative predictive biomarkers for response to anti-epidermal growth factor receptor (EGFR) therapy and carry distinct prognostic implications^4,5^. These observations raise the question of whether similar molecular heterogeneity influences outcomes and therapeutic sensitivity in earlier-stage disease treated with curative intent.

Approximately one-third of CRC patients present with stage III disease, and up to one-fifth of those with stage II disease exhibit high-risk features associated with a higher likelihood of recurrence after curative surgery^6^. Despite complete resection, a considerable proportion will experience relapse, reflecting the presence of residual micrometastatic disease^7^. Adjuvant chemotherapy remains the standard of care for these patients, aiming to eradicate microscopic tumor deposits and improve long-term outcomes^7,8^. Fluoropyrimidine monotherapy, most commonly with 5-fluorouracil (5-FU) or capecitabine, has served as the foundational regimen for decades, while the addition of oxaliplatin, forming the FOLFOX or CAPOX combinations, has yielded incremental survival gains in stage III disease^9,10^. Addition of oxaliplatin improved 5-year disease-free survival (DFS) by roughly 5 to 7 percentage points and overall survival (OS) by 2 to 3 percentage points compared with fluoropyrimidine alone^11^. Among patients with high-risk stage II disease, however, the absolute benefit was up to 3 percentage points, while cumulative neurotoxicity remains a substantial clinical burden^12^. These limitations have motivated efforts to identify molecular determinants of treatment benefit that could inform a more selective use of oxaliplatin-based therapy.

In the adjuvant setting, the predictive associations for *KRAS* have been inconsistent: a single-center study reported that adjuvant chemotherapy, largely oxaliplatin based, improved 3-year DFS, primarily among *KRAS*-mutated tumors, whereas no significant treatment interaction was observed with *KRAS* mutation in pooled analyses of randomized clinical trials^13–15^. For *BRAF*, multiple analyses confirm persistent poor prognosis despite adjuvant therapy, including FOLFOX, but do not demonstrate a reliable modification of chemotherapy benefit^16–20^. Notably, many prior trials contrasted 5-FU and leucovorin versus irinotecan or evaluated the addition of biologics such as cetuximab, rather than directly testing the incremental value of oxaliplatin over fluoropyrimidine alone^9,14,19^. In addition, most studies combined stages II and III without granular high-risk definitions^17,19^. These design differences leave unresolved whether *KRAS* or *BRAF* mutation status modifies the benefit of adding oxaliplatin to a fluoropyrimidine backbone in contemporary stage III and high-risk stage II practice.

To address these gaps, we analyzed data from the DACHS study, a large, population-based cohort with detailed molecular characterization and long-term follow-up. Our objectives were: first, to evaluate the association of *KRAS* and *BRAF*^*V600E*^ mutation status with recurrence-free survival (RFS) and OS among patients with stage III and high-risk stage II (T4N0) CRC; and second, to determine whether these mutations modify the survival benefit of oxaliplatin-based adjuvant chemotherapy compared with fluoropyrimidine alone. By integrating population-based outcomes with molecular tumor profiling, this study aims to investigate the potential for risk stratification and identify biomarker-defined subgroups that may guide the personalization of adjuvant chemotherapy decisions in early-stage CRC.

## Results

### Study population

**Fig. 1:**
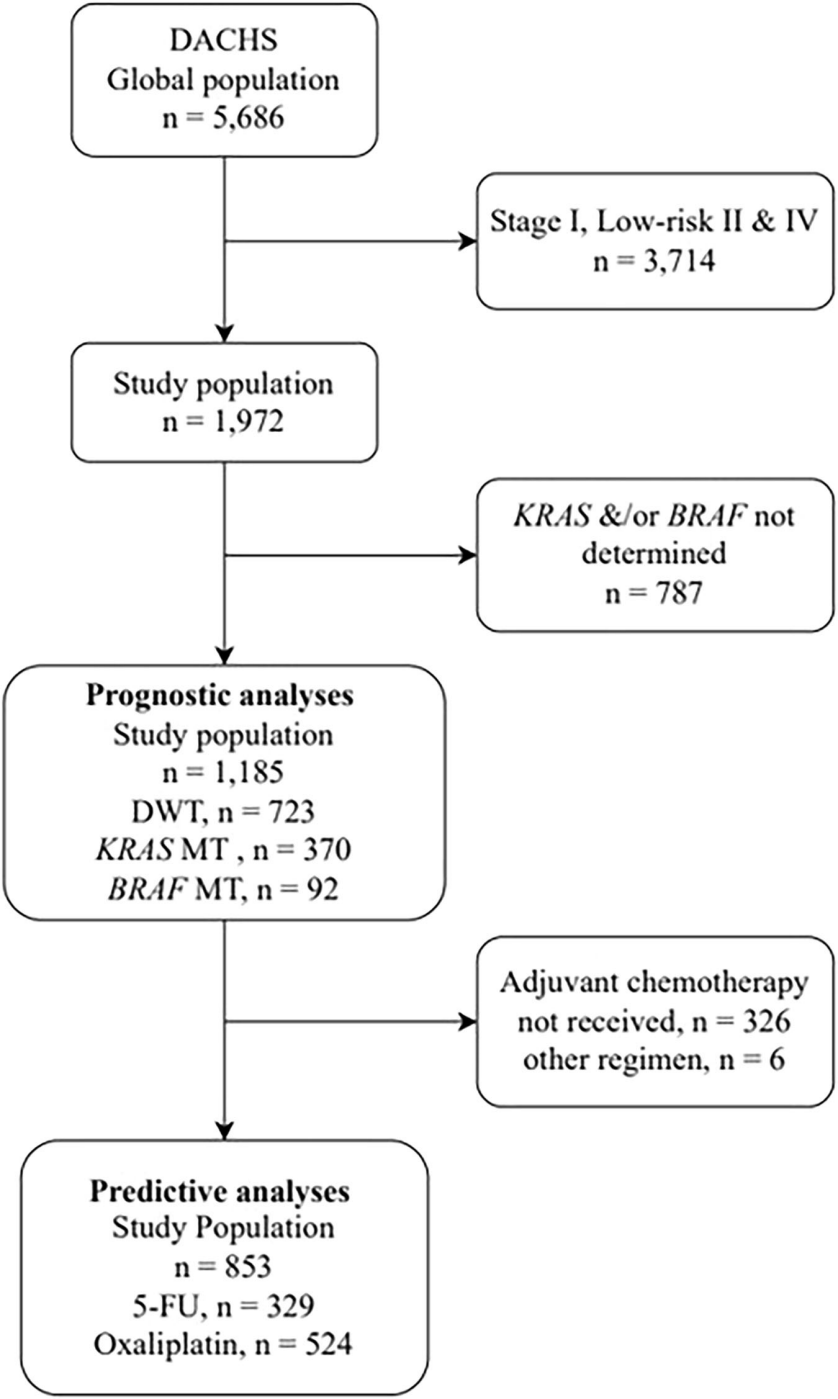
Study population selection for prognostic and predictive analyses. DWT double wild-type, MT mutated.

Between 2003 and 2021, 5686 patients were recruited in the DACHS cohort, 1972 were in stage III and high-risk stage II, and 1185 had data on the mutation status and were eligible for prognostic analyses (Fig. 1). Adjuvant chemotherapy data were available for all eligible patients, of which 853 received adjuvant 5-FU or oxaliplatin-based therapy and were included in the predictive analyses.

### Baseline characteristics

Baseline characteristics of the study population according to *KRAS* and *BRAF* mutation status are shown in Table 1. Overall, 31% of tumors harbored a *KRAS* mutation, 8% a *BRAF* mutation, and 61% were DWT. *BRAF* MT tumors were more common among older patients, women, and right-sided and microsatellite instability (MSI)-high cancers, and were more frequently poorly differentiated. *KRAS* MT tumors, in contrast, were typically left-sided and microsatellite stable (MSS). The distribution of T and N stage was similar across mutation groups, and most patients (~90%) had stage III disease. Approximately 72% of the patients received adjuvant chemotherapy, most commonly an oxaliplatin-based regimen, with no meaningful difference in treatment distribution by mutation status.

**Table 1:** Demographic and baseline clinical characteristics of patients with stage III and high-risk stage II colorectal cancer in DACHS

Variable	DWT (*N* = 723)	*KRAS* MT (*N* = 370)	*BRAF* MT (*N* = 92)	*p* value
Age, median [y, IQR]	69 [61, 76]	70 [61, 76]	74 [64, 82]	0.0002
Age <50 y, *n* (%)	49 (6.8)	23 (6.2)	3 (3.3)	0.69
Female sex, *n* (%)	294 (40.7)	163 (44.1)	62 (67.4)	<0.0001
BMI, median [IQR]	27 [24, 29]	26 [24, 29]	27 [24, 30]	0.49
Smoking status, *n* (%)				0.20
Non-smoker	329 (45.5)	189 (51.1)	50 (54.3)	
Former	287 (39.7)	129 (34.9)	27 (29.3)	
Current	107 (14.8)	52 (14.1)	15 (16.3)	
Charlson comorbidity index, *n* (%)				0.10
CC0	293 (40.5)	153 (41.4)	24 (26.1)	
CC1	189 (26.1)	94 (25.4)	29 (31.5)	
CC2	241 (33.3)	123 (33.2)	39 (42.4)	
Diabetes, *n* (%)	140 (19.4)	63 (17.0)	23 (25.0)	0.21
Regular aspirin use, *n* (%)	144 (19.9)	80 (21.6)	22 (23.9)	0.60
Regular statin use, *n* (%)	103 (14.2)	54 (14.6)	15 (16.3)	0.87
Neoadjuvant therapy, *n* (%)	128 (17.7)	38 (10.3)	2 (2.2)	<0.001
Tumor location, *n* (%)				<0.0001
Right	199 (27.5)	151 (40.8)	74 (80.4)	
Left	196 (27.1)	84 (22.7)	8 (8.7)	
Rectum	328 (45.4)	135 (36.5)	10 (10.9)	
Stage III, *n* (%)	644 (89.1)	344 (93.0)	78 (84.8)	0.47
T stage, *n* (%)				0.57
T1	11 (1.5)	5 (1.4)	2 (2.2)	
T2	77 (10.7)	32 (8.6)	8 (8.7)	
T3	451 (62.4)	251 (67.8)	55 (59.8)	
T4	184 (25.4)	82 (22.2)	27 (29.3)	
N stage, *n* (%)				0.14
N0	79 (10.9)	26 (7.0)	14 (15.2)	
N1	426 (58.9)	219 (59.2)	48 (52.2)	
N2	218 (30.2)	125 (33.8)	30 (32.6)	
Tumor grade, *n* (%)				<0.0001
G1	11 (1.5)	2 (0.5)	0 (0.0)	
G2	514 (71.1)	271 (73.2)	36 (39.1)	
G3	198 (27.3)	97 (26.2)	56 (60.9)	
MSI-high, *n* (%)	51 (7.1)	25 (6.8)	49 (53.3)	<0.0001
Adjuvant chemotherapy, *n* (%)	524 (72.5)	276 (74.6)	59 (64.1)	0.13
Regimen, *n* (%)				0.33
5-FU	198 (27.4)	111 (30.0)	20 (21.7)	
Oxaliplatin	322 (44.5)	163 (44.1)	39 (42.4)	
Other	4 (0.5)	2 (0.5)	0 (0.0)	

*y* years, *IQR* interquartile range, *MSI* microsatellite instability, *MSS* microsatellite stable, *MT* mutated, *CC* Charlson comorbidity, *DWT* double wild-type.

### Prognostic analyses

The median follow-up of the study population was 10.1 years (95% confidence interval (CI), 10–10.2 years), 10.1 (95% CI, 10–10.2 years), and 10 years (95% CI, 9.2–10.6 years) for patients with DWT, *KRAS* MT, and *BRAF* MT tumors, respectively. During this follow-up time, 427 recurrences or CRC–related deaths and 569 deaths from any cause were reported.

Patients with *BRAF* MT tumors were associated with shorter RFS and OS, while *KRAS* MT had similar survival compared with those DWT tumors (Supplementary Fig. 1A, B). In multivariable Cox models, *KRAS* MT was not associated with a significant difference in RFS or OS compared with DWT tumors (RFS hazard ratio (HR) = 1.07; 95% CI, 0.87–1.34; OS HR = 1.12; 95% CI, 0.93–1.34). In contrast, RFS and OS were significantly shorter in patients with *BRAF* MT tumors compared with patients with DWT tumors (RFS HR = 1.71; 95% CI, 1.14–2.56; OS HR = 1.67; 95% CI, 1.18–2.37) (Table 2).

**Table 2:** Association between mutation status and survival in stage III and high-risk stage II colorectal cancer patients

Variable		Recurrence-free survival		Overall survival	
		Total/event (*n*)	HR (95%CI)	Total/event (*n*)	HR (95%CI)
Mutation	DWT	717/240	1	723/325	1
	*KRAS* MT	366/127	1.07 (0.87–1.34)	370/191	1.12 (0.93–1.34)
	*BRAF* MT	91/34	1.71 (1.14–2.56)	92/53	1.67 (1.18–2.37)

Models adjusted for age, sex, tumor location, T & N stage, tumor grade, microsatellite instability status, adjuvant chemotherapy, Charlson comorbidity index (CCI), body mass index (BMI), smoking, and use of aspirin and statins.

*DWT* double wild type, *MT* mutated, *HR* hazard ratio, *CI* confidence interval.

### Predictive analyses

To account for non-randomized treatment allocation, we performed propensity score weighting, where propensity score weighting achieved excellent covariate balance between treatment groups, with standardized mean differences (SMDs) for all covariates below 0.1 after weighting (Supplementary Fig. 2). The distribution of propensity scores demonstrated adequate overlap between treatment groups (Supplementary Fig. 3A, B), supporting the assumption of common support required for valid weighted comparisons.

In weighted Cox models including an interaction between mutation status and treatment regimen, the overall interaction was statistically significant for both OS (*p*_interaction_ = 0.007) and RFS (*p*_interaction_ = 0.049), indicating that the survival association of oxaliplatin-based chemotherapy differed by molecular subgroup (Supplementary Fig. 4A–F).

Formal pairwise comparisons of the treatment associations (oxaliplatin-based therapy vs fluoropyrimidine monotherapy) across mutation subgroups confirmed significant differences in survival associations (Supplementary Table S1). For OS, the treatment association in *KRAS*-mutated tumors differed significantly from that in both DWT tumors (*p* = 0.039) and *BRAF*-mutated tumors (*p* = 0.003), while *BRAF* and DWT tumors also differed (*p* = 0.049). For RFS, a similar pattern was observed, with a significant difference between *KRAS*-mutated and *BRAF*-mutated tumors (*p* = 0.021), whereas the remaining comparisons were not statistically significant despite similar directional patterns (*BRAF*-mutated vs DWT, *p* = 0.10; *KRAS*-mutated vs DWT, *p* = 0.12).

**Fig. 2:**
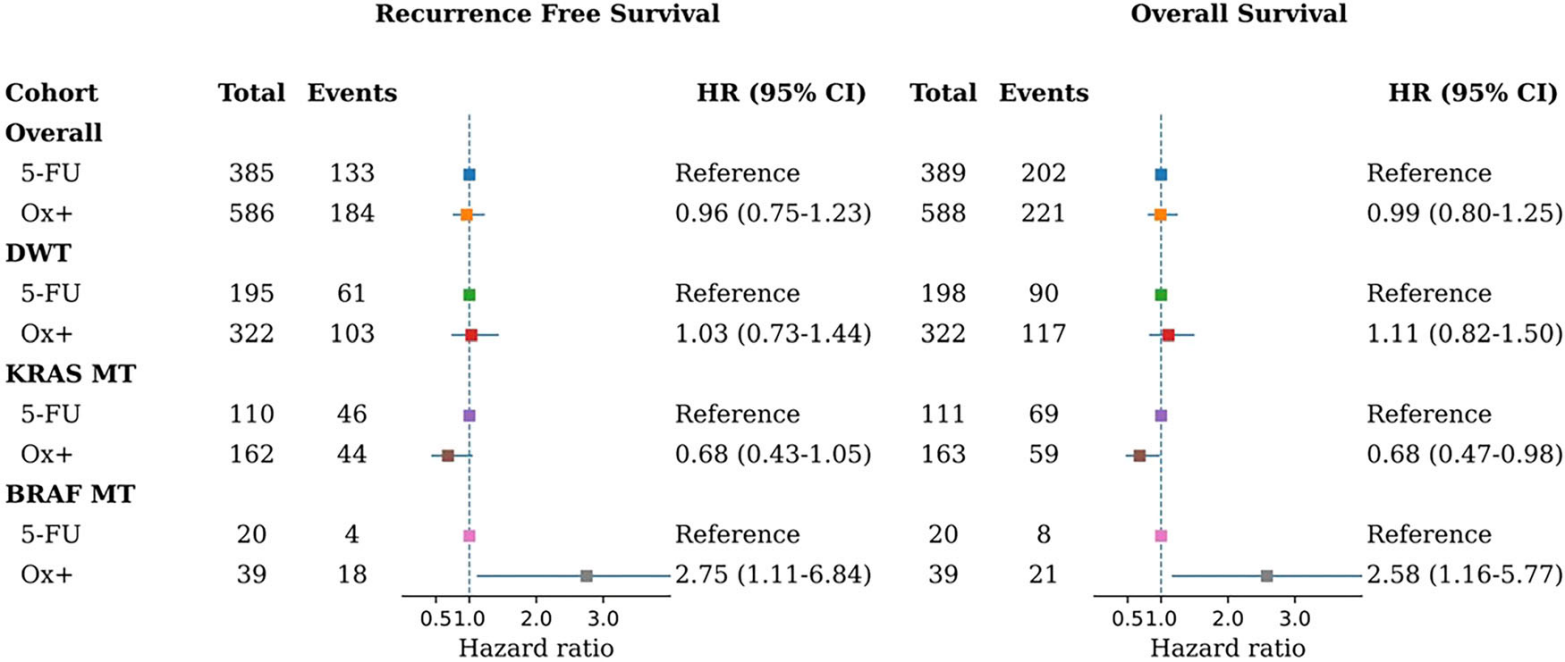
Association between adjuvant chemotherapy regimens and survival in stage III and high-risk stage II colorectal cancer patients. Ox+ oxaliplatin combination therapy, 5FU fluoropyrimidine monotherapy, HR hazard ratio, CI confidence interval, MT mutated. Hazard ratios were estimated using multivariable Cox proportional hazards models adjusted for age, sex, Charlson comorbidity index, tumor location, T and N stage, tumor grade, microsatellite instability status, body mass index (BMI), smoking, and use of aspirin and statins. Corresponding *p* values for within-subgroup comparisons (oxaliplatin vs fluoropyrimidine) were: overall cohort (RFS *p* = 0.75; OS *p* = 0.94), DWT cohort (RFS *p* = 0.87; OS *p* = 0.50), *KRAS*-mutated cohort (RFS *p* = 0.08; OS *p* = 0.04), and *BRAF*-mutated cohort (RFS *p* = 0.03; OS *p* = 0.02). The interaction between mutation status and treatment regimen was statistically significant (OS *p* = 0.007; RFS *p* = 0.049).

On performing stratified analyses by mutation status, among patients with DWT tumors, oxaliplatin-based therapy was not associated with prolonged survival compared with fluoropyrimidine monotherapy (OS: HR = 1.11; 95% CI, 0.82–1.50; RFS: HR = 1.03; 95% CI, 0.73–1.44). In contrast, patients with *KRAS* MT tumors experienced improved outcomes with oxaliplatin (OS: HR = 0.68; 95% CI, 0.47–0.98; RFS: HR = 0.68; 95% CI, 0.43–1.05), whereas those with *BRAF* MT tumors showed worse survival after oxaliplatin-based chemotherapy (OS: HR = 2.58; 95% CI, 1.16–5.77; RFS: HR = 2.75; 95% CI, 1.11–6.84) (Fig. 2).

Sensitivity analyses adjusting for neoadjuvant therapy showed no meaningful differences in results for OS, with a similar directional pattern but reduced precision for RFS (Supplementary Table S2).

## Discussion

In this large population-based cohort with over a decade of follow-up, we found that the effectiveness of adjuvant chemotherapy differed according to molecular subtype, with a significant interaction between mutation status and treatment regimen. Patients with *KRAS*-mutated tumors experienced improved outcomes with oxaliplatin-based combination therapy compared with fluoropyrimidine monotherapy, while those with *BRAF*^*V600E*^-mutated tumors showed no evidence of benefit and even a potential association with poorer survival. No meaningful difference in survival was observed between the two regimens among patients with DWT tumors. These findings highlight that the prognostic and predictive relevance of RAS–RAF pathway alterations extends beyond the metastatic setting and may have implications for refining adjuvant treatment selection in early-stage CRC.

Our finding that *BRAF*^*V600E*^ mutation is a strong adverse prognostic factor for both RFS and OS is consistent with extensive prior evidence from clinical trials and pooled analyses. The PETACC-8 and CALGB 89803 trials demonstrated substantially poorer outcomes for *BRAF*-mutated compared with *BRAF*-wild-type stage III colon cancers, independent of treatment regimen or microsatellite status^14,19,21^. Likewise, meta-analyses of randomized adjuvant trials confirmed *BRAF*^*V600E*^ as a reproducible marker of poor prognosis across stage II and III disease^15,16^. Our results reinforce these observations in a population-based setting, highlighting that *BRAF* mutation remains a key determinant of unfavorable tumor biology even outside the trial context.

The absence of a significant prognostic association for *KRAS* mutation in our cohort is broadly consistent with several prior studies but contrasts with others reporting a modest adverse survival association. In CALGB 89803 and single center restrospective studies, no difference in DFS, RFS, or OS was observed between *KRAS*-mutated and wild-type tumors, and the QUASAR trial similarly reported only weak, inconsistent associations between *KRAS* status and recurrence risk^14,22,23^. However, meta-analyses and pooled trial datasets reported modestly worse OS and DFS for *KRAS*-mutated versus wild-type tumors after adjustment for MSI status, while PETACC-8 identified a poorer prognosis restricted to MSS tumors. Similarly, the recent ACCENT/IDEA pooled analysis revealed codon-specific heterogeneity, with the G12C variant showing the most unfavorable outcome^15,18,19^. Differences in analytic granularity, particularly a lack of stratification by specific *KRAS* codons (G12C, G12D, G12V, G13D), and the broader molecular and clinical diversity of the population-based cohort compared with selective clinical trial populations, may account for these differences.

Our observation that patients with *KRAS*-mutated tumors experienced improved survival with oxaliplatin-based adjuvant chemotherapy compared with fluoropyrimidine monotherapy suggests a possible predictive signal for treatment benefit in this subgroup. Importantly, pairwise contrasts confirmed that the association of oxaliplatin differed significantly between *KRAS*-mutated and *BRAF*-mutated tumors, with a trend toward better outcomes in *KRAS*-mutated compared with DWT cancers, indicating that *KRAS* mutant tumors may drive much of the overall treatment heterogeneity observed. Major randomized trials, including CALGB 89803 and PETACC-8, found no statistically significant interaction between *KRAS* status and treatment arm, although these trials primarily evaluated regimens containing irinotecan or biologic agents rather than directly isolating the addition of oxaliplatin to a 5-FU backbone^14,19,24^. In contrast, the study on early-stage CRC and smaller retrospective series in metastatic disease have similarly suggested greater platinum sensitivity among *KRAS* mutant tumors^13,25^. Mechanistic data lend support to this pattern: *KRAS*-mutated cells exhibit impaired upregulation of ERCC1, a key DNA repair enzyme, following platinum exposure, resulting in increased susceptibility to oxaliplatin-induced cytotoxicity^26^. While the present findings require confirmation in independent datasets, they support the hypothesis that *KRAS* mutation may identify a subgroup of early-stage CRC with greater susceptibility to oxaliplatin-based chemotherapy relative to other molecular profiles.

Our data suggest a possible pattern of diminished benefit from oxaliplatin-based adjuvant chemotherapy among patients with *BRAF*^*V600E*^-mutated tumors compared with other molecular subgroups. In stratified analyses, outcomes appeared worse with oxaliplatin than with fluoropyrimidine alone; however, pairwise contrasts indicated that the differential treatment associations were most pronounced between *BRAF*- and *KRAS*-mutated tumors and less distinct when compared with DWT disease. Previously, the MOSAIC 10-year update and PETACC-8 studies found *BRAF* mutation to be an adverse prognostic factor regardless of regimen, but did not demonstrate a treatment interaction or evidence of adverse outcomes following oxaliplatin therapy^15,16,19,20^. The divergence from these findings could reflect limited sample size of *BRAF*-mutated cases, residual confounding despite propensity score weighting, or biological heterogeneity, given that *BRAF* mutant tumors are typically right-sided and MSI-high, features associated with reduced responsiveness to cytotoxic therapy^11,27^. Adjusting for tumor location and MSI status did not meaningfully alter our results, though precision remained limited. Mechanistically, sustained MAPK activation and microenvironmental factors such as stromal remodeling, immune exclusion, and altered DNA damage responses may attenuate oxaliplatin efficacy in *BRAF* mutant CRCs^3,28,29^. Given that current guidelines recommend oxaliplatin-based adjuvant therapy for stage III CRC irrespective of *BRAF* status, these associations should be interpreted as hypothesis-generating pending validation in larger biomarker-stratified cohorts^7,8^.

Among patients with DWT tumors, oxaliplatin-based adjuvant chemotherapy was not associated with longer RFS or OS compared with fluoropyrimidine monotherapy, and pairwise contrasts showed no significant difference in oxaliplatin associations between the DWT and either *KRAS* or *BRAF*-mutated groups for RFS. This suggests that the modest absolute survival benefit of oxaliplatin in unselected stage III populations, typically a 5–7% improvement in DFS, may not extend uniformly across all molecular subtypes^9,11^. If validated, this finding could have practical implications for treatment de-escalation, supporting omission or shortening of oxaliplatin exposure in biomarker-defined, low-benefit patients to minimize cumulative neurotoxicity. Such a precision-based approach would complement current strategies, which emphasize individualized adjuvant therapy duration according to patient and tumor risk profiles^18,30^.

This study has several strengths. The large, population-based study design captures treatment patterns representative of real-world practice. The extended follow-up period (median, 10 years) allows for mature assessment of both RFS and OS, ensuring stability of long-term estimates. Comprehensive molecular characterization within a well-defined cohort enabled simultaneous evaluation of *KRAS* and *BRAF*^*V600E*^ mutations for both prognostic and predictive associations with consideration of other relevant molecular and clinical factors. Use of propensity score overlap weighting addressed confounding by indication in the non-randomized treatment comparison, achieving excellent covariate balance between treatment groups. Unlike most prior studies that contrasted heterogeneous regimens, this analysis directly compared fluoropyrimidine monotherapy with the addition of oxaliplatin, addressing a clinically relevant question in contemporary adjuvant practice. Additional adjustment for neoadjuvant therapy, which was largely confined to rectal cancer, did not materially alter the OS findings and showed similar directional associations for RFS, suggesting that the observed heterogeneity was not explained solely by preoperative treatment. Finally, the adequate sample size and substantial number of outcome events provided sufficient power for the primary prognostic analyses and permitted exploratory survival associations with treatment regimens across molecular strata.

Several limitations should also be acknowledged. First, as an observational study, residual confounding cannot be excluded despite rigorous adjustment using propensity score weighting and verification of covariate balance. Second, the *BRAF*^*V600E*^ mutant subgroup was relatively small (approximately 8% of the cohort), particularly within the fluoropyrimidine-only arm, resulting in wide CIs and reduced precision of predictive estimates. Third, *KRAS* mutation analyses were limited to exon 2 without codon-level distinction, precluding assessment of potential subtype-specific heterogeneity. Fourth, modest heterogeneity existed in chemotherapy regimens, with both 5-FU and capecitabine used as fluoropyrimidines and both FOLFOX and CAPOX included in the oxaliplatin-based group, although these reflect real-world practice patterns. Data on treatments received after disease recurrence were not available in the present study. Post-relapse treatments, particularly anti-EGFR therapies, whose efficacy depends on *RAS/BRAF* status, could differentially influence OS across mutation subgroups, potentially confounding the interpretation of our findings. However, the consistent direction of the mutation-treatment interaction for RFS, an outcome unaffected by post-relapse therapy, suggests that the observed associations are not solely attributable to downstream treatment differences. Future studies with comprehensive treatment trajectory data are warranted to further clarify this issue. Finally, the long accrual period (2003–2021) spans advances in surgical techniques, perioperative care, and adjuvant management that could influence outcomes.

In conclusion, our findings may have important implications for the clinical management and future trial design of early-stage CRC. Routine testing for *KRAS* and *BRAF* mutations should be incorporated into standard evaluation of stage III and high-risk stage II disease, not only for prognostic stratification but also for further studies on refining adjuvant treatment selection^18^. If validated, a pragmatic treatment approach could involve reserving oxaliplatin-based chemotherapy for *KRAS*-mutated tumors, while considering fluoropyrimidine monotherapy or alternative strategies for patients with *BRAF* MT or DWT tumors. In parallel, studies for *BRAF*^*V600E*^-mutated early-stage disease should prioritize evaluation of rational targeted combinations, such as *BRAF* and MEK inhibition^3,31^. Lastly, integrating *KRAS* and *BRAF* mutations with circulating tumor DNA or tumor cells may yield composite risk models for individualized adjuvant therapy selection in CRC^11^. Prospective biomarker-interaction trials are warranted to confirm these observations and to establish the clinical utility of *KRAS* and *BRAF* mutations for optimizing adjuvant treatment selection in early-stage CRC.

## Methods

### Study population

We used data from the Darmkrebs: Chancen der Verhütung durch Screening (DACHS) study, a population-based case-control and patient cohort study conducted in southwest Germany. The design and data collection procedures have been described previously^32,33^. In brief, since 2003, patients with a first CRC diagnosis have been consecutively recruited from 22 hospitals serving the study catchment area and are followed longitudinally through standardized questionnaires, medical record reviews, and linkage with regional cancer registries and mortality databases. This study was performed in accordance with the Declaration of Helsinki. The study was approved by the ethics committees of the University of Heidelberg (310/2001) and the Medical Councils of Baden-Württemberg and Rhineland-Palatinate, and all participants provided written informed consent. This research was conducted according to the STROBE (Strengthening the Reporting of Observational Studies in Epidemiology) guidelines^34^.

For the present analysis, we included patients with pathologically confirmed stage III or high-risk stage II (T4N0) colorectal adenocarcinoma who underwent curative (R0) surgical resection and had information on adjuvant chemotherapy and *KRAS*/*BRAF* mutation status. Patients with distant metastases at diagnosis, prior invasive cancer (except nonmelanoma skin cancer), and unknown adjuvant treatment were excluded. A subset of patients, predominantly those with rectal cancer, received neoadjuvant therapy before surgery and were retained in the analytic cohort. Targeted therapies and immunotherapy were not part of standard adjuvant treatment during the study period and were not administered in this setting.

Of all eligible patients meeting inclusion criteria, those without follow-up information or with missing exposure or molecular data were excluded prior to analysis; no additional loss to follow-up occurred after cohort entry beyond administrative censoring at last contact. Information on adjuvant chemotherapy regimen, start and completion dates, and dose adjustments was abstracted from hospital discharge summaries and oncology records.

### Molecular profiling

MSI was evaluated using a panel of mononucleotide markers (BAT25, BAT26, and CAT25)^35^. The *BRAF*^*V600E*^ [*c.1799T* > *A (p.V600Glu)*] mutation was identified through two complementary approaches. In 52% of cases, immunohistochemical staining was performed on tissue microarray sections and independently reviewed by two experienced pathologists (HB, MK). The remaining 48% were analyzed by Sanger sequencing of exon 15^35^. The frequency of detected alterations was comparable between the two methods, indicating consistent assay performance^35^. *KRAS* mutation testing was carried out on extracted tumor DNA using either single-strand conformation polymorphism analysis (48%) or Sanger sequencing targeting exon 2 (codons 12 and 13) (52%)^35^. Mutations in other *KRAS* exons (exons 3 and 4) were not assessed.

### Exposure assessment

Patients were categorized into double wild-type for both *KRAS* and *BRAF*^*V600E*^ (DWT), *KRAS* mutated (*KRAS* MT), and *BRAF*^*V600E*^ mutated (*BRAF* MT), where DWT was considered the reference group. Adjuvant chemotherapy was categorized as fluoropyrimidine monotherapy (5-FU or capecitabine) or oxaliplatin-based combination therapy (FOLFOX or CAPOX). A small number of patients who received other regimens (e.g., irinotecan-based or experimental protocols) were analyzed descriptively but were excluded from the primary comparative analyses. Adjuvant chemotherapy regimens were not randomly assigned but were determined by treating physicians according to clinical practice at the time of diagnosis, with physicians blinded to the outcomes.

### Outcomes

The outcomes for these analyses were RFS and OS. RFS was defined as the time from diagnosis to the first occurrence of tumor recurrence, distant metastasis, or CRC-related death, whichever occurred first, with patients censored at the last follow-up if no event occurred. OS was measured from diagnosis to death from any cause, and survivors were censored at the date of last confirmed vital status.

### Statistical analyses

Baseline characteristics were summarized as medians (interquartile ranges, IQRs) for continuous variables and as frequencies and percentages for categorical variables, with differences between groups assessed using the *χ*² test or Wilcoxon rank-sum test, as appropriate. Survival analyses were conducted using the Kaplan–Meier method and log-rank test.

For prognostic analyses, Cox proportional hazards models were used to estimate HRs and 95% CIs for the association of *KRAS* and *BRAF* mutation status with RFS and OS, adjusted for age, sex, tumor location, T and N stage, grade, MSI status, adjuvant chemotherapy, Charlson comorbidity index (CCI), body mass index (BMI), smoking, and use of aspirin and statins.

For predictive analyses, we examined whether *KRAS* or *BRAF* status modified the association between adjuvant chemotherapy regimen and survival, excluding those who did not receive chemotherapy. To address potential confounding due to non-random treatment allocation, we applied propensity score weighting. Propensity scores were estimated from a logistic regression model including tumor stage, grade, location, MSI status, comorbidities, BMI, smoking, and medication use. Overlap weights were derived as the primary estimand, and balance between treatment groups was evaluated using SMDs before and after weighting, with SMDs <0.1 indicating acceptable balance. Weighted Cox proportional hazards models were then fitted to estimate HRs for the oxaliplatin versus 5-FU groups within mutation strata, using robust (sandwich) variance estimators to account for weighting. Prespecified formal interaction terms (treatment × mutation status) were included to test for modification of association by *KRAS* and *BRAF* (1).

1 $$h(t)={h}_{0}(t)\times exp({\beta }_{1}{\cdot }Regimen+{\beta }_{2}{\cdot }KRAS+{\beta }_{3}{\cdot }BRAF+{\beta }_{4}\cdot (Regimen\times KRAS)+{\beta }_{5}\cdot (Regimen\times BRAF))$$

where Regimen is an indicator for oxaliplatin-based combination therapy versus fluoropyrimidine monotherapy (reference), *KRAS* and *BRAF* are indicator variables for *KRAS* mutant and *BRAF*^*V600E*^ mutant tumors, respectively, with DWT as the reference category, and the product terms (Regimen × *KRAS*) and (Regimen × *BRAF*) capture the interaction between treatment regimen and mutation status. Confounders were addressed through propensity score overlap weighting. A global Wald test on the interaction terms (*β*₄ and *β*₅ jointly equal to zero) was used to assess whether the association between treatment regimen and survival differed across mutation subgroups.

The analytic cohort included adult patients (≥30 years) of both sexes, with age at diagnosis modeled as a continuous covariate in all adjusted analyses. Sex was included in all multivariable and propensity score models, and analyses were adjusted for sex to account for potential differences in CRC prognosis and treatment patterns. All prognostic and predictive analyses, including treatment-by-mutation interaction tests, were prespecified prior to data analysis. Sensitivity analyses additionally adjusted for neoadjuvant therapy in the propensity score and Cox models.

Model assumptions of proportional hazards were verified using Schoenfeld residuals. Given the focused nature of the hypotheses tested and the limited number of prespecified interaction contrasts, no formal adjustment for multiple comparisons was applied. Formal a priori power calculations were not performed as the current study was based on a population-based observational study using all available eligible cases. All statistical tests were two-sided, and *p* values < 0.05 were considered statistically significant. Analyses were performed using R software (version 4.3.0; R Foundation for Statistical Computing, Vienna, Austria RRID:SCR_001905), using *survival* (RRID:SCR_021137), *WeightIt* (RRID:SCR_023649), and *tableone* (RRID:SCR_015640) packages.

## Supplementary information

Supplementary Information

## Data Availability

The datasets generated and/or analyzed during the current study are not publicly available due to data privacy protection laws. However, grouped data can be shared on a reasonable request to the corresponding author.
