# Supplementary material for: KRAS and BRAF mutations modify adjuvant chemotherapy outcomes in early stage colorectal cancer
Source: NPJ Precis Oncol. 2026 May 20;10:186. doi: 10.1038/s41698-026-01494-y (PMC13190747; doi:10.1038/s41698-026-01494-y)
Supplement: Supplementary file 1 — Supplementary Information [file 41698_2026_1494_MOESM1_ESM.pdf]

***KRAS* and *BRAF*^{V600E} mutations altered predictive effectiveness of adjuvant chemotherapy in stage III and high-risk stage II colorectal cancer: A prospective cohort study**

Durgesh Wankhede; Mary Jose Urruchua Rodriguez; Dominic Edelmann; Matthias Kloor; Hendrik Bläker; Alexander Brobeil; Wilfried Roth; Hermann Brenner; Michael Hoffmeister

Supplemental File

Description		Page no.
Supplementary Figure S1	Prognostic associations of KRAS and BRAF mutations with recurrence-free and overall survival.	2
Supplementary Figure S2	Standardized mean differences of baseline covariates before and after weighting by different propensity score methods	3
Supplementary Figure S3	Propensity score distribution for adjuvant chemotherapy regimen A. fluoropyrimidine monotherapy, B. Oxaliplatin combination therapy.	4
Supplementary Figure S4	Predictive associations of oxaliplatin-based versus fluoropyrimidine monotherapy on recurrence-free and overall survival, stratified by tumor mutation status.	5
Supplementary Table S1	Pairwise contrasts of treatment associations across mutation subgroups for survival in stage III and high-risk stage II colorectal cancer	7
Supplementary Table S2	Sensitivity analyses of the association between KRAS and BRAF mutation status, adjuvant chemotherapy regimen, and survival in stage III and high-risk stage II colorectal cancer, with additional adjustment for neoadjuvant therapy	8

Supplementary Figure 1

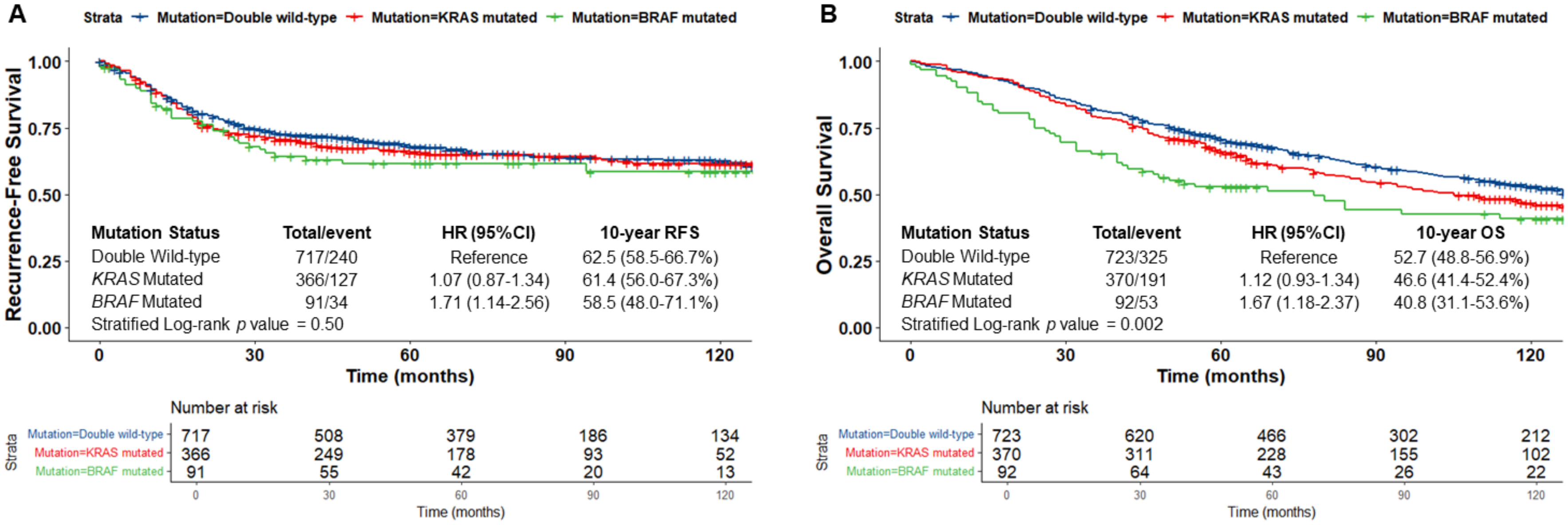

Supplementary Figure 1. Prognostic associations of *KRAS* and *BRAF* mutations with recurrence-free and overall survival. (A) Recurrence-free survival (RFS) and (B) overall survival (OS) among patients with stage III and high-risk stage II colorectal cancer, stratified by tumor mutation status. Survival curves are based on Kaplan–Meier estimates; 10-year survival probabilities are shown for each group. Hazard ratios (HRs) and 95% confidence intervals (CIs) were derived from multivariable Cox regression models adjusted for age, sex, tumor T and N categories, histologic grade, tumor location, microsatellite status, adjuvant chemotherapy regimen, Charlson comorbidity index, body mass index, a history of diabetes, aspirin and statin use.

Supplementary Figure 2

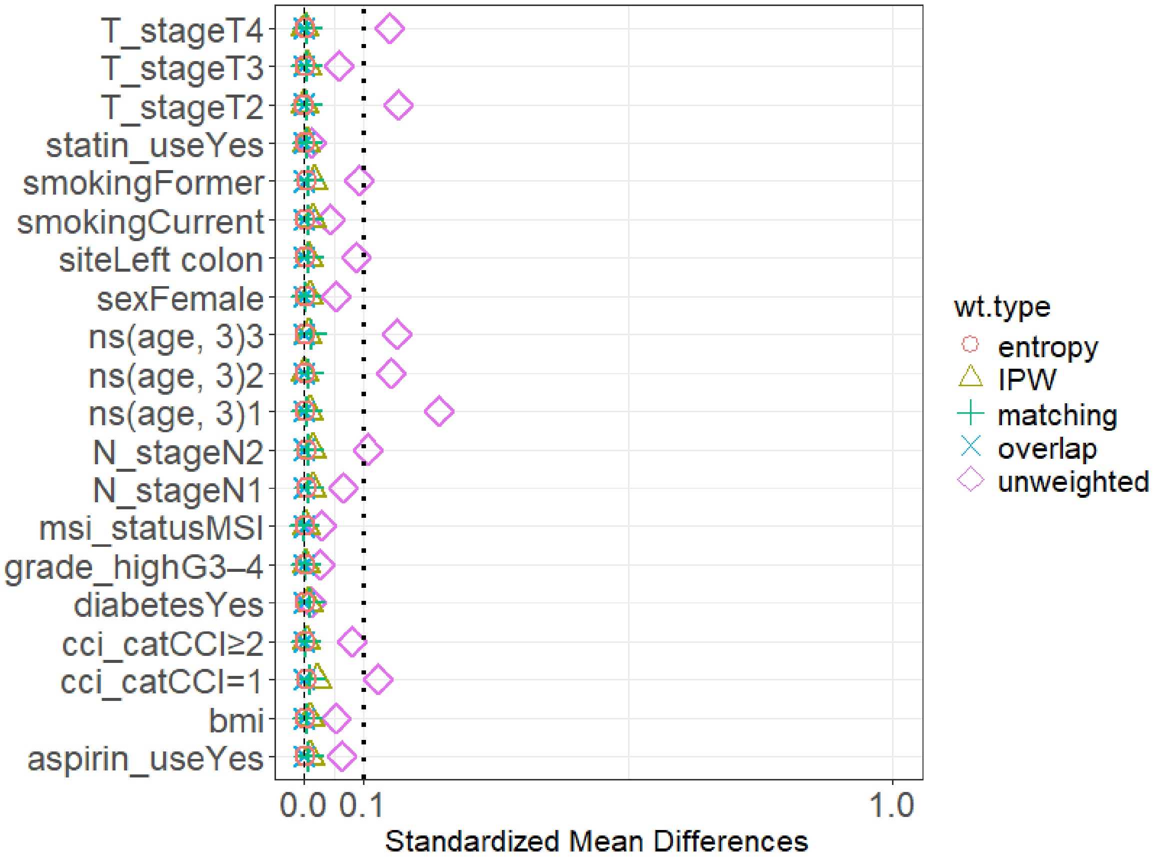

Supplementary Figure S2. Standardized mean differences of baseline covariates before and after weighting by different propensity score methods. Covariates included age (modeled using natural cubic splines with three degrees of freedom), sex, tumor site, T and N stage, histologic grade, microsatellite instability (MSI) status, body mass index, smoking status, Charlson comorbidity index, diabetes, and use of aspirin and statins.

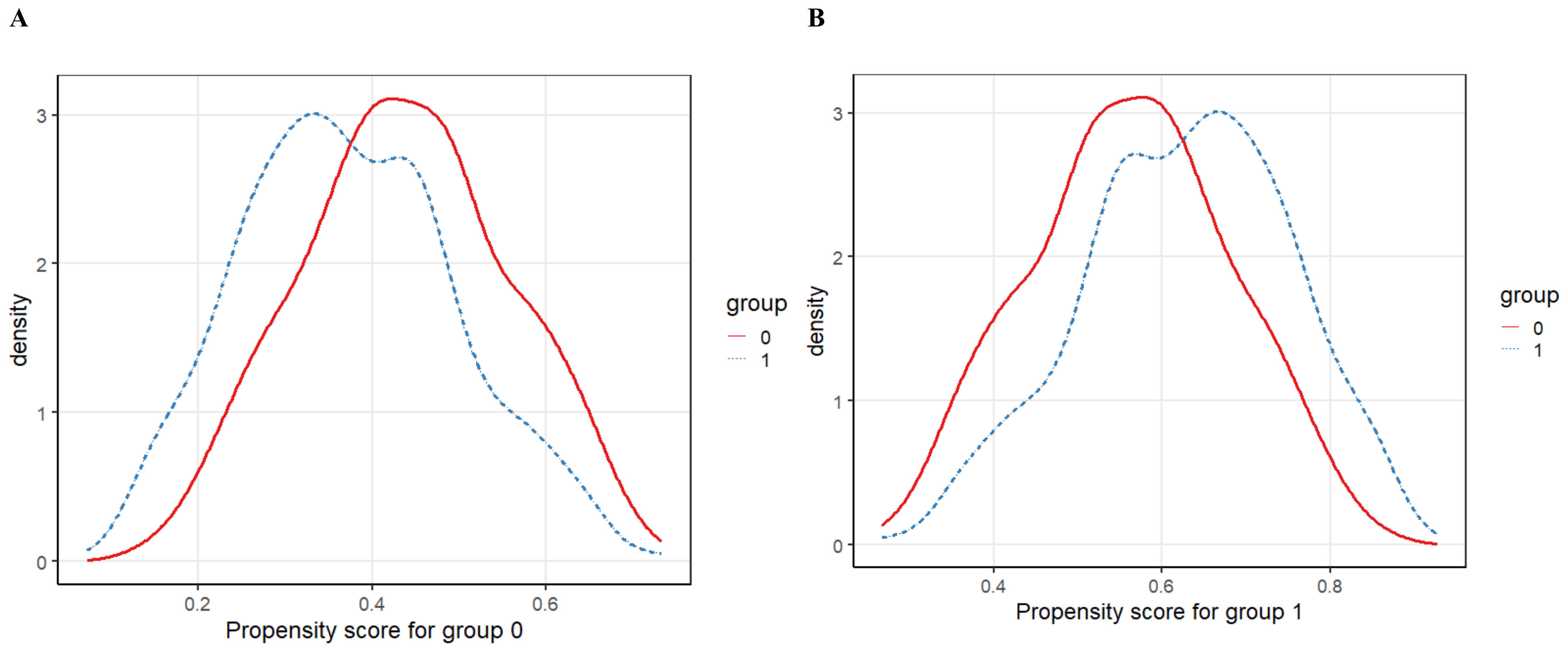

Supplementaty Figure S3. Propensity score distribution for adjuvant chemotherapy regimen A. fluoropyrimidine monotherapy, B. Oxaliplatin combination therapy. Group 0, fluoropyrimidine monotherapy; group 1, Oxaliplatin combination therapy.

Supplementary Figure 4

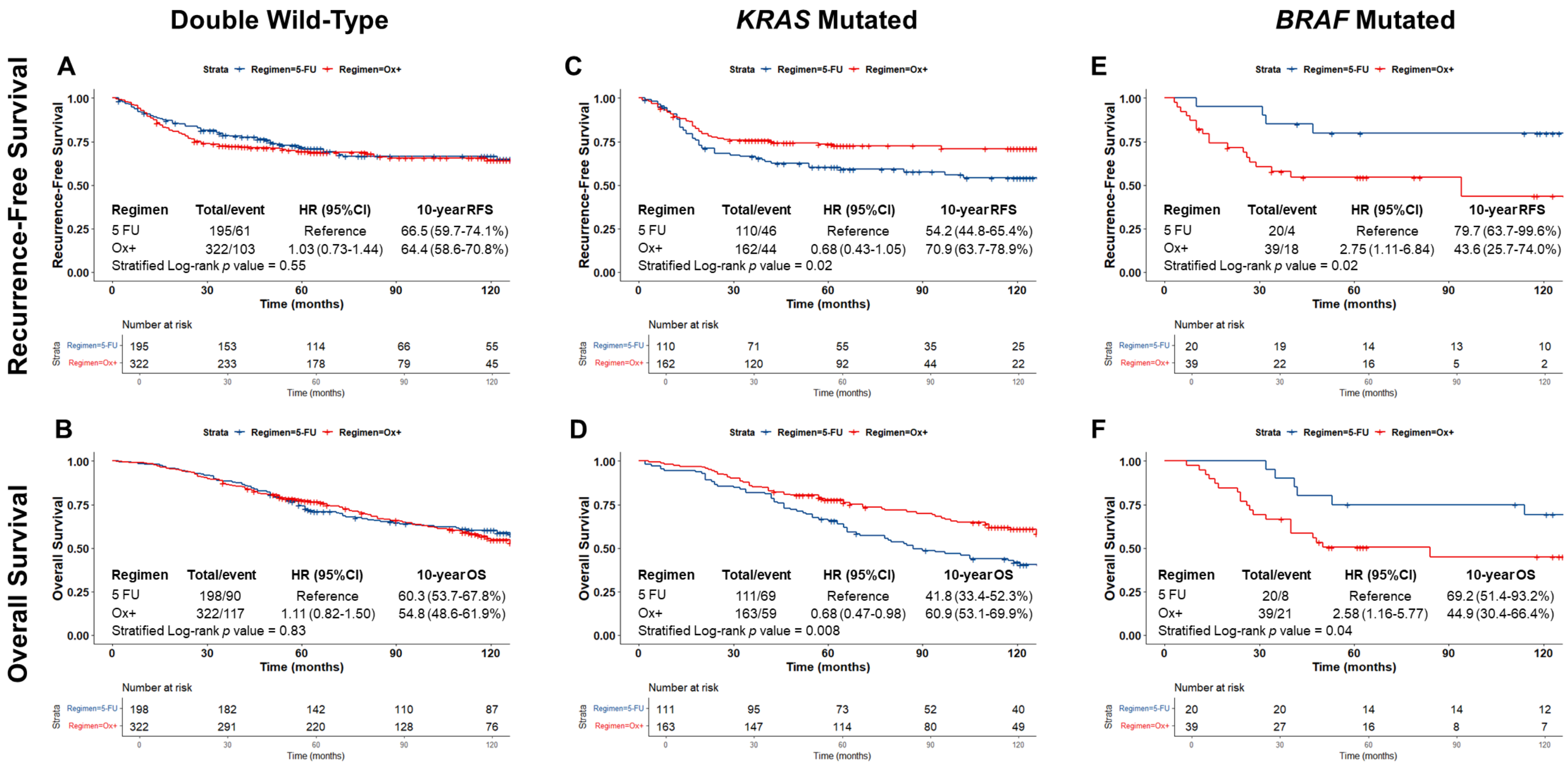

Supplementary Figure 4. Predictive associations of oxaliplatin-based versus fluoropyrimidine monotherapy on recurrence-free and overall survival, stratified by tumor mutation status. Kaplan–Meier curves show recurrence-free survival (RFS; Panels A,C, & E) and overall survival (OS; Panels B, D, & F) among patients treated with fluoropyrimidine alone (blue) or an oxaliplatin-containing regimen (red), stratified by mutation subgroup: double wild-type (A, D), KRAS mutated (B, E), and BRAF mutated (C, F). Hazard ratios (HRs) and 95% confidence

intervals (CIs) were derived from propensity score overlap weighted Cox models adjusted for age, sex, tumor T and N categories, histologic grade, tumor location, microsatellite status, Charlson comorbidity index, body mass index, a history of diabetes, aspirin and statin use.

Supplementary Table S1. Pairwise contrasts of treatment associations across mutation subgroups for survival in stage III and high-risk stage II colorectal cancer

Mutation-Regimen Pairs	Recurrence-Free Survival		Overall Survival	
	Ratio	*p* value	Ratio	*p* value
((5-FU) / (Ox+) *BRAF* MT) / ((5-FU) / (Ox+) *KRAS* MT)	0.255	0.02	0.253	0.003
((5-FU) / (Ox+) *BRAF* MT) / ((5-FU) / (Ox+) DWT)	0.394	0.10	0.419	0.04
((5-FU) / (Ox+) *KRAS* MT) / ((5-FU) / (Ox+) DWT)	1.54	0.12	1.65	0.03

Ratios represent the relative difference in the oxaliplatin treatment association between molecular subgroups, expressed as the ratio of hazard ratios (RHR) on the log-hazard scale. Within each subgroup, the term “(5-FU) / (Ox+)” denotes the hazard ratio comparing fluoropyrimidine monotherapy with oxaliplatin-based therapy. Ratio values <1 indicate a less favorable oxaliplatin effect in the numerator subgroup compared with the denominator subgroup. *p* values are based on Wald tests derived from linear contrasts of interaction model coefficients. 5-FU, flouropyrimidine monotherapy, Ox+, oxaliplatin combination therapy, MT, mutated, DWT, double wild type.

Supplementary Table S2. Sensitivity analyses of the association between *KRAS* and *BRAF* mutation status, adjuvant chemotherapy regimen, and survival in stage III and high-risk stage II colorectal cancer, with additional adjustment for neoadjuvant therapy

Analysis	Variable	HR (95% CI)
**Prognostic OS**	*KRAS* mutated vs double wild-type	1.13 (0.94–1.35)
	BRAF mutated vs double wild-type	1.63 (1.15–2.32)
**Prognostic RFS**	*KRAS* mutated vs double wild-type	1.07 (0.86–1.34)
	BRAF mutated vs double wild-type	1.71 (1.14–2.56)
**Predictive OS**	*KRAS* mutated × oxaliplatin (vs 5-FU)	0.61 (0.38–0.97)
	BRAF mutated × oxaliplatin (vs 5-FU)	2.39 (1.00–5.69)
**Predictive RFS**	*KRAS* mutated × oxaliplatin (vs 5-FU)	0.64 (0.37–1.12)
	BRAF mutated × oxaliplatin (vs 5-FU)	2.53 (0.82-7.79)

Hazard ratios (HRs) and 95% confidence intervals (CIs) were estimated using multivariable Cox proportional hazards models. Prognostic analyses evaluated the association between mutation status and survival outcomes. Predictive analyses assessed the interaction between mutation status and adjuvant chemotherapy regimen (oxaliplatin-based vs fluoropyrimidine monotherapy). All models were additionally adjusted for neoadjuvant therapy, in addition to, age, sex, tumor location, T & N stage, tumor grade, microsatellite instability status, adjuvant chemotherapy, Charlson comorbidity index (CCI), body mass index (BMI), smoking, and use of aspirin and statins.
